# Anisotropic MOF-on-MOF Growth of Isostructural Multilayer Metal–Organic Framework Heterostructures

**DOI:** 10.34133/2021/9854946

**Published:** 2021-11-16

**Authors:** Zhida Gu, Wenlei Zhang, Ting Pan, Yu Shen, Peishan Qin, Peng Zhang, Xiaohan Li, Liwei Liu, Linjie Li, Yu Fu, Weina Zhang, Fengwei Huo

**Affiliations:** ^1^College of Science, Northeastern University, Shenyang 100819, China; ^2^Key Laboratory of Flexible Electronics (KLOFE), Institute of Advanced Materials (IAM), Nanjing Tech University (NanjingTech), Nanjing 211800, China

## Abstract

Isostructural MOFs with similar crystallographic parameter are easily available for MOF-on-MOF growth and possible to form core–shell structure by isotropic growth. However, due to well-matched cell lattice, selective growth in isostructural MOF heterostructures remains a great challenge for engineering atypical MOF heterostructures. Herein, an anisotropic MOF-on-MOF growth strategy was developed to structure a range of multilayer sandwich-like ZIF-L heterostructures via stacking isostructural ZIF-L-Zn and ZIF-L-Co alternately with three-, five-, seven-, and more layer structures. Moreover, these heterostructures with highly designable feature were fantastic precursors for fabricating derivatives with tunable magnetic and catalytic properties. Such strategy explores a novel way of achieving anisotropic MOF-on-MOF growth between isostructural MOFs and opens up new horizons for regulating the properties by MOF modular assembly in versatile functional nanocomposites.

## 1. Introduction

Metal–organic frameworks (MOFs) are a type of porous crystalline materials which are coordinated by metal ions and organic ligands with highly ordered and periodic network. They have attracted tremendous attention due to their intriguing properties such as large surface area, tailorable cavity, flexible structure, and component diversification [1, 2]. These features bring about a wide variety of MOFs, and most of them own distinguished advantages. Compared with individual MOFs, MOF heterostructures integrating two or more MOFs have exhibited better performances or even novel properties in catalysis [3–5], gas separation [6, 7], sensing [8], drug delivery [9], and energy storage [10, 11], mainly owing to their designable interface [12, 13], hierarchical pore structure [14], and synergetic effect between individual MOFs [15]. In addition, the limitations of individual MOFs can be compensated by forming the MOF heterostructures. Therefore, construction of MOF heterostructures has attracted wide attentions and become new recent research interest [16].

To date, many methods have been developed to fabricate MOF heterostructures, such as epitaxial growth method (EGM) [17], surfactant-assisted method [18], postsynthetic modification (PSM) [19, 20], ligand exchange method [21], and kinetic control method [22]. These strategies make great contributions to the enrichment of MOF heterostructures. Generally, core–shell type MOF heterostructures are dominant products, in which the secondary MOF is uniformly coated on template MOF by isotropic growth [22]. However, to satisfy multifarious demands in properties and applications, orientation growth of secondary MOF becomes key to engineering MOF heterostructures. Thus, atypical MOF heterostructures with growth adjustability and structural designability show more opportunities than the typical core–shell structure. Recently, anisotropic MOF-on-MOF growth strategy has been proven to be effective in achieving well-designed MOF heterostructures, in which the growth rate and direction of the secondary MOF can be controlled to render selective growth on specific vertexes, edges, or surfaces of the template MOF especially via appropriate small cell lattice mismatch [5, 23, 24]. Specifically, the slightly mismatched lattice could induce In-MIL-88B to grow selectively on Fe-MIL-88B by utilizing the size variation between InO_6_ and FeO_6_ octahedra for layer-type hybrid heterostructure [25]. Induced by ligand variation between naphthalene-1,4-dicarboxylic acid and benzene dicarboxylic acid, anisotropic growth of MOF-NDC on template In-MIL-68 was achieved for novel MOF heterostructure [26]. The position of (110) crystal surface in MIL-125 controlled the secondary ZIF-8 to grow selectively on the corner or side surface of the template MOF for preparing two types of MIL-125@ZIF-8 heterostructures [27, 28]. However, identifying appropriate small cell lattice mismatch between different types of MOFs for anisotropic MOF-on-MOF growth is quite challenging, which limits the development of MOF heterostructures to some extent. On the contrary, isostructural MOFs with well-matched cell lattices not only are easily available, but also possess high compatibility between the template and the secondary MOFs due to the identical ligand length and topological structure. Usually, well-matched cell lattices result in the production of isotropic growth for core–shell type MOF heterostructures. Actually, there is a broad consensus among researchers that during the growth of crystal materials, intrinsic discrepancy of different crystal surfaces in one crystal material can also be used to induce different growth rates on these surfaces [29]. If such intrinsic crystal surface discrepancy in MOFs could be utilized to induce the anisotropic MOF-on-MOF growth between available isostructural MOFs, there will be more opportunities for developing anisotropic MOF-on-MOF growth between available isostructural MOFs.

**Figure 1:**
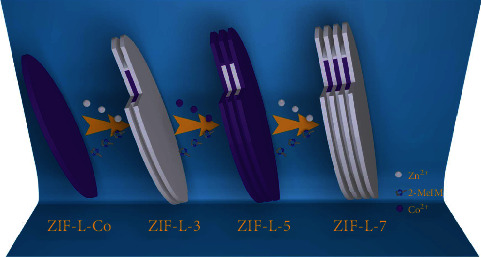
Schematic illustration of the multilayer ZIF-L heterostructures.

Herein, a facile anisotropic MOF-on-MOF growth strategy was developed to prepare a range of multilayer MOF heterostructures by utilizing the intrinsic crystal surface discrepancy of two isostructural MOFs. To demonstrate this concept, two kinds of zeolitic imidazolate frameworks (ZIFs), ZIF-L-Co and ZIF-L-Zn, which are coordinated by cobalt ions or zinc ions with 2-methylimidazole (2-MeIM), were utilized alternately as templates for anisotropic MOF-on-MOF growth. These two isostructural ZIF-L with similar two-dimensional leaf-like morphologies, layered network topologies, and discrepant crystal surfaces decided their promising possibility for anisotropic MOF-on-MOF growth. Via multiple utilization of such growth, a range of multilayer sandwich-like ZIF-L heterostructures were able to be achieved with three, five, seven, and even more layers (Figure 1). During the growth process, it was considered that the different growth rates and orders of the secondary MOF on the template MOF were possibly caused by the discrepancies of coordination modes, relaxation effects, and surface energies between crystal surfaces in one ZIF-L. These well-organized multilayer ZIF-L heterostructures can serve as precursors for fabricating ZIF-L heterostructure derivatives, which not only inherited the controllable layer-by-layer structures from the parent ZIF-L heterostructures, but also obtained new hierarchical porosity with designed space distribution of cobalt-based nanoparticles. Moreover, the as-prepared ZIF-L heterostructure derivatives showed excellent performances in the fields of magnetics and selective catalysis. Based on these results, the facile strategy of anisotropic epitaxial growth between isostructural MOFs is expected to be a general method to explore more potential templates for anisotropic MOF-on-MOF growth as well as more novel MOF heterostructures for well-suited applications.

## 2. Results

### 2.1. Synthesis and Structure Characterization of Multilayer ZIF-L Heterostructures

To realize the concept, we used presynthesized ZIF-L-Co as the template, in which zinc ions are coordinated with 2-MeIM, and accordingly generated two new ZIF-L-Zn layers on template ZIF-L-Co, forming a sandwich-like three-layer ZIF-L-Zn/ZIF-L-Co/ZIF-L-Zn heterostructure. Subsequently, cobalt ions and 2-MeIM were added and coordinated on the abovementioned heterostructures. Similarly, two ZIF-L-Co layers were formed, yielding a five-layer alternating heterostructure. Each time the process was repeated, two new ZIF-L layers would be constructed for advanced ZIF-L heterostructures. Herein, these three-, five-, seven-, and X-layer ZIF-L heterostructures were named as ZIF-L-X, where X represented the total layer number.

**Figure 2:**
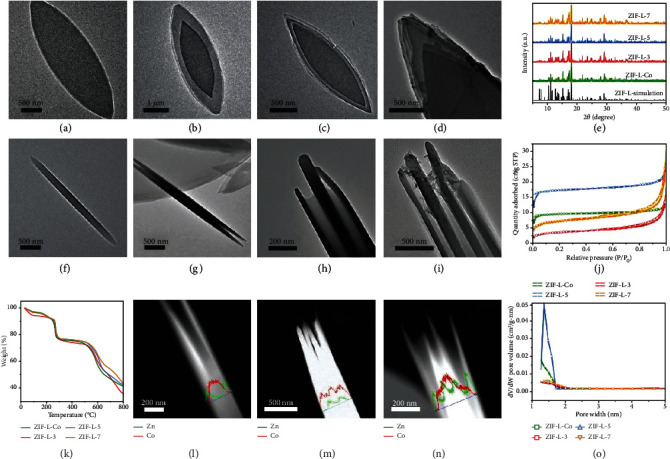
Characterizations of the multilayer ZIF-L heterostructures. TEM images of (a, f) ZIF-L-Co, (b, g) ZIF-L-3, (c, h) ZIF-L-5, and (d, i) ZIF-L-7. (e) PXRD patterns, (j) nitrogen adsorption–desorption isotherms, (o) pore size distribution image, and (k) thermogravimetric curves of the multilayer ZIF-L heterostructures. The line scan of (l) ZIF-L-3, (m) ZIF-L-5, and (n) ZIF-L-7.

The morphology, crystal structure, thermostability, and porosity of ZIL-L heterostructures were further characterized by transmission electron microscopy (TEM), scanning electron microscopy (SEM), powder X-ray diffraction (PXRD), thermogravimetric analysis (TGA), and nitrogen adsorption–desorption measurements, respectively. As shown in the TEM images, ZIF-L-Co presented a leaf-like shape with length of ~4 *μ*m, width of ~2 *μ*m (Figure 2(a) and S1(a), Supplementary Materials), and thickness of ~120 nm (Figure 2(f)), which corresponded to the reported ZIF-L-Co structure [30]. ZIF-L-3 (Figure S1(c)) showed a concentric leaf-like morphology from the top view (Figure 2(b)) and a three-layer sandwich-like structure from the lateral view (Figure 2(g)). We considered that ZIF-L-3 was composed of a template ZIF-L-Co layer with ~120 nm thickness and two ZIF-L-Zn layers with 100 nm thickness, which were confirmed by the elemental distribution (Figure S2) and the line scan image (Figure 2(l)) of ZIF-L-3 with Co elements dominating the middle layer and Zn elements occupying the other two layers. Similarly, ZIF-L-5 (Figure S1(d)) and ZIF-L-7 (Figure S1(e)) exhibited the five- (Figures 2(c) and 2(h)) and seven-layer (Figures 2(d) and 2(i)) structures, respectively, and were composed of ZIF-L-Zn and ZIF-L-Co layers in an alternate way, as further demonstrated by the line scan images (Figures 2(m) and 2(n)). Compared to the XRD spectrum of simulated ZIF-L [31], no significant crystal peak loss was detected in XRD patterns of the multilayer ZIF-L heterostructures, confirming the phase purity and framework stability during alternate growth of ZIF-L-Zn and ZIF-L-Co (Figure 2(e)). The TGA spectra of the multilayer ZIF-L heterostructures (Figure 2(k)) under nitrogen showed that they all had similar three-stage temperature decline at 100−200°C (solvent removal), 260−270°C (2-MeIM evaporation), and 530−600°C (carbonization loss), demonstrating their similar thermostability [31]. The Brunauer–Emmett–Teller (BET) surface areas from ZIF-L-Co to ZIF-L-7 were 38.3, 12.4, 71.8, and 25.56 m^2^·g^−1^. In addition, they had similar nitrogen adsorption–desorption isotherms (Figure 2(j)) and pore size distribution (Figure 2(o)) [32].

### 2.2. Anisotropic MOF-on-MOF Growth Mechanism of ZIF-L Heterostructures

**Figure 3:**
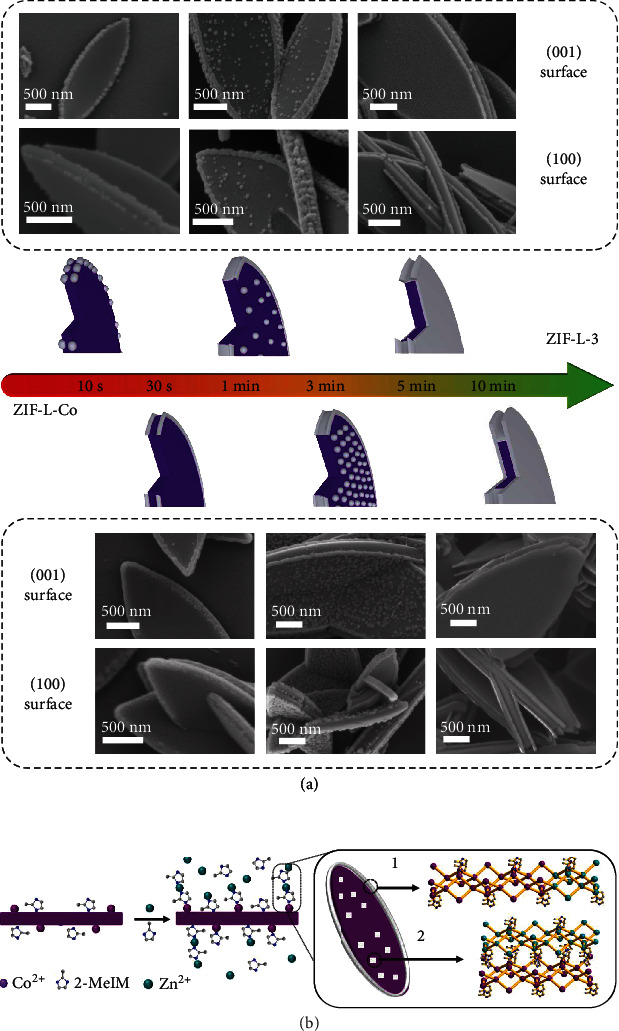
Synthetic process and mechanism of ZIF-L-3. (a) Schematic diagram and SEM images of ZIF-L-3 formation process from two views. (b) Coordination on different crystal surfaces for the growth of ZIF-L-3.

**Figure 4:**
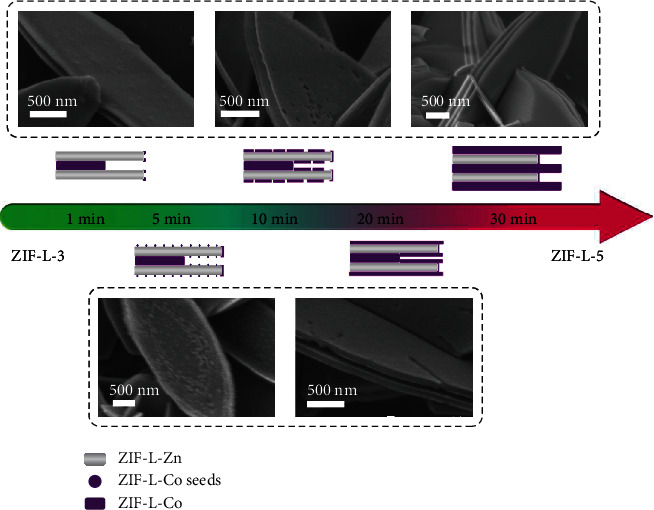
Schematic diagram and SEM images of ZIF-L-5 formation process.

To further understand the formation mechanism, TEM (Figure 4S) and SEM (Figure 3(a)) were employed to monitor the anisotropic MOF-on-MOF growth process of multilayer ZIF-L heterostructures at different times. From ZIF-L-Co to ZIF-L-3, it was observed that the ZIF-L-Zn crystal seeds started to grow on the edges between leaf-like surfaces and lateral surfaces of the template ZIF-L-Co at 10 s and formed into loops at 30 s. With the growth of these crystal seeds, they touched with each other and provided a thin cover on the lateral surface, while new crystal seeds appeared on the leaf-like surface at 1 min. At 3 min, the lateral surface of the template ZIF-L-Co became smooth while the leaf-like surface exhibited more crystal seeds. Along with the growth time prolonging, the crystal seeds on the leaf-like surface touched each other and grew together, resulting in the formation of two new thin ZIF-L-Zn layers on the template at 5 min. At 10 min, these new layers became larger and thicker until ZIF-L-3 was synthesized. In a typical ZIF-L-Zn crystal, six bridging 2-MeIM ligands were bound with two Zn ions for a large hexagon, while four bridging 2-MeIM ligands were bound with two Zn ions for a smaller parallelogram [31]. Two-dimensional layer networks were created by interconnecting hexagons and parallelograms. ZIF-L-Zn was synthesized by stacking layer networks and stabilized by the hydrogen bond of 2-MeIM between the layers (Figure S5) [31]. Therefore, such coordination and stacking led surfaces in orientations to exhibit discrepant surface properties, resulting in selective growth of the secondary MOF on different surfaces (Figure 3(b)). Two types of coordination modes were involved in ZIF-L-Zn including coordination bonds on the leaf-like surface and hydrogen bonds on the lateral surface [33]. Generally, the preferable sequence of bond breaking would be hydrogen bond, coordination bond, and covalent bond in the MOFs [33]. According to the calculation, the surface energy of leaf-like (001) surface was about one hundredth of that of the other surface (including (100) surface) [33]. It meant that the lateral surfaces with higher surface energy would grow preferentially than the leaf-like surfaces with low surface energy. This conclusion is basically consistent with the previous observation. At the beginning of structuring the ZIF-L-3, the free metal ions and ligands preferred to coordinate on the template instead of renucleating in solution. Especially, crystal seeds preferentially grew on the crystal edges between the two surfaces than any other surfaces at 10 s probably due to the lower coordination number [34]. Furthermore, the crystal seed fusion at 30 s and 1 min on the lateral surface should be contributed to the unique relaxation effect of ZIF-L which was more significant to the lateral surface than the leaf-like surface [33]. At the same time, such relaxation effect allowed the crystal interfacial structure to relax and release the strain energy, resulting in the gradual decrease in surface energy on the edge of ZIF-L-Co along with the crystal seed coordination [35]. Afterwards, the leaf-like surface became more attractive for crystal seed to grow at 3 min. Similarly, the relaxation effect allowed the crystal seed to fuse on the leaf-like surface which induced the formation of new ZIF-L-Zn layers at 5 min. In a word, the process from ZIF-L-Co to ZIF-L-3 inspired that the intrinsic crystal surface discrepancies could induce the anisotropic MOF-on-MOF growth between the isostructural MOFs.

Meanwhile, according to the intrinsic crystal surface discrepancy theory, both ZIF-L-Co and ZIF-L-Zn have potentials as the initial template MOF. If it is true, these two isostructural ZIF-L-Co and ZIF-L-Zn could be the modular building blocks for coding domain distribution and sequencing within a specific ZIF-L heterostructure just like playing Lego. Therefore, presynthesized ZIF-L-Zn was tested to be the template for anisotropic growth of ZIF-L-Co. A ZIF-L-Zn-based three-layer heterostructure was synthesized and exhibited similar three-layer morphology (Figure S6) but different element distribution (Figure S7) to ZIF-L-3. SEM (Figure S8) and TEM (Figure S9) images exhibited the growth and fusion processes of crystal seeds at 10 s, the formation of new ZIF-L-Co layers at 1 min, and the enlarging and thickening processes of these layers at 10 min, which were similar to the formation process of ZIF-L-3. The template potential of both ZIF-L-Co and ZIF-L-Zn could provide more designability by modular assembly for diversified multilayer ZIF-L heterostructures.

Afterwards, the process from ZIF-L-3 to ZIF-L-5 was also monitored by SEM (Figure 4). Initially at 1 min, the edge of the template ZIF-L-3 became brighter in the image, which was considered as edge growth just like the first process from ZIF-L-Co to ZIF-L-3. Afterwards, many ZIF-L-Co seeds grew on the leaf-like surface of template ZIF-L-3 at 5 min. With the growth and mergence of these ZIF-L-Co seeds, new thin layers were formed and covered both the external and internal leaf-like surfaces at 10 min. The newly formed layers became thicker and larger and even outstretched the edges at 20 min. Surprisingly, the ZIF-L-5 was observed to be synthesized at 30 min. Therefore, what is the middle layer in the formation process of ZIF-L-5 becomes a very interesting question. A very possible explanation is that two internal ZIF-L-Co layers merged with each other by continuous thickening and became the middle layer of ZIF-L-5 in a similar way as the crystal seed fusion for new ZIF-L-Zn layers at 5 min in ZIF-L-3 formation. Furthermore, the above process provided a theoretical support for constructing multilayer alternating MOF-on-MOF heterostructures.

### 2.3. Synthesis and Structure Characterization of ZIF-L Heterostructure Derivatives

**Figure 5:**
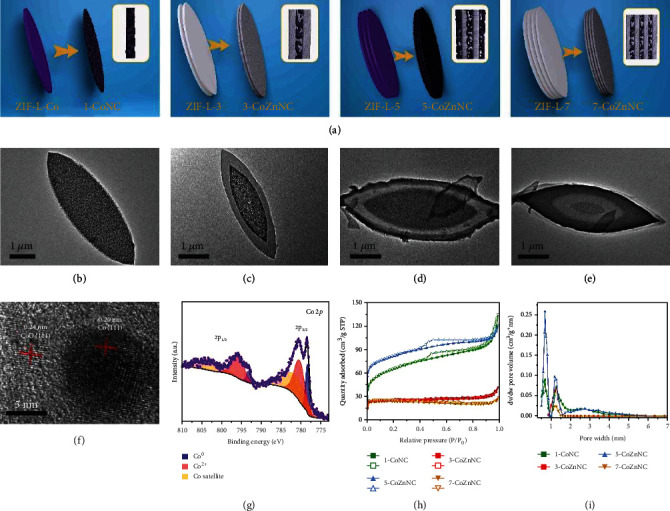
Characterization of the multilayer ZIF-L heterostructure derivatives. (a) Schematic illustration of the formation and TEM images of (b) 1-CoNC, (c) 3-CoZnNC, (d) 5-CoZnNC, and (e) 7-CoZnNC via thermal treatment. (f) HRTEM image and (g) Co 2*p* XPS spectra for 1-CoNC. (h) Nitrogen adsorption–desorption isotherms and (i) pore size distribution of the multilayer ZIF-L heterostructure derivatives.

Metal or metal oxide-doped functional porous carbon-based composites have shown fascinating prospects as photocatalysts [36], electrocatalysts [37, 38], supercapacitors [39], etc. Benefitting from the structural designability and component flexibility, multilayer ZIF-L heterostructures could serve as precursors of multilayer ZIF-L heterostructure derivatives for remarkable performances. Here, a range of multilayer ZIF-L heterostructures were calcinated to multilayer ZIF-L heterostructure derivatives at 600°C for 2 h (Figure 5(a) and S10). The morphology, compositions, and porous structure of these multilayer ZIF-L heterostructure derivatives can be further characterized by TEM, X-ray photoelectron spectroscopy (XPS), and BET analysis. According to the TEM images, all derivatives maintained the unique hierarchical layer structures of their pristine ZIF-L heterostructures. Specifically, ZIF-L-Co derivative (Figure 5(b)) with leaf-like morphology exhibited uniform distribution of nanoparticles with size of ~10 nm. ZIF-L-3 derivative (Figure 5(c)) exhibited three-layer hierarchical structure including an inner layer and two outer layers. The inner ZIF-L-Co derivative layer held nanoparticles with size of ~8 nm, while the outer ZIF-L-Zn derivative layers showed no nanoparticles. Similar to ZIF-L-3 derivative, ZIF-L-5 derivative (Figure 5(d)) and ZIF-L-7 derivative (Figure 5(e)) presented corresponding multilayer derivative structures which were composed of ZIF-L-Zn and ZIF-L-Co derivative layers. According to the XPS data of ZIF-L-Co (Figure S12(a)) and ZIF-L-3 (Figure S12(d)) derivatives, their C 1s spectra exhibited similar four peaks located at 284.8, 285.9, 287.1, and 289.0 eV which corresponded to C−C, C−N, C−O, and C=O bonds, respectively (Figure S12(b) and S122(e)). In their N 1*s* spectra, similar two peaks which located at 398.9 and 400.8 eV were assigned to pyridinic and pyrrolic nitrogen, respectively, which proved the nitrogen doping in the carbon frameworks (Figure S12(c) and S12(f)) [40]. The Zn 2*p* spectrum of ZIF-L-3 derivative exhibited quite a strong signal peak at 1021.7 eV, which suggested that the zinc was doped in ZIF-L-3 derivative (Figure S12(h)). At the same time, the Co 2*p* spectrum of ZIF-L-Co derivative was comprised of two main peaks located at 778.4 and 780.6 eV, which corresponded to Co^0^ and Co^2+^ (Figure 5(g)). However, the peak signal of ZIF-L-3 derivative was too weak to reflect the chemical state of cobalt (Figure S12(g)), probably because the outer layer cover of ZIF-L-Zn derivative restricted the XPS detection. In addition, the high-resolution TEM (HRTEM) image of ZIF-L-Co derivative (Figure 5(f)) showed the crystalline Co with a clear 0.20 nm lattice spacing which was ascribed to their (111) surface [41] and the crystalline CoO with a clear 0.24 nm lattice spacing which was consistent with their (111) surface [42], implying the nanoparticles were composed of Co and CoO. Therefore, it was recognized that these multilayer ZIF-L heterostructure derivatives were composed of cobalt and nitrogen-doped carbon or cobalt, zinc, and nitrogen-doped carbon, denoted as 1-CoNC, 3-CoZnNC, 5-CoZnNC, and 7-CoZnNC, respectively.

These ZIF-L heterostructure derivatives with diverse layer numbers and sequences exhibited different structural properties. As illustrated in the nitrogen adsorption–desorption isotherms of these derivatives (Figure 5(h)), the appearance of hysteresis loops implied the presence of mesopores in 1-CoNC and 5-CoZnNC and the absence of mesopores in 3-CoZnNC and 7-CoZnNC. As calculated, the BET surface areas of 1-CoNC and 5-CoZnNC (234.9 m^2^·g^−1^ and 296.4 m^2^·g^−1^) were apparently higher than those of 3-CoZnNC and 7-CoZnNC (114.4 m^2^·g^−1^ and 100.4 m^2^·g^−1^). Furthermore, 1-CoNC and 5-CoZnNC showed peaks at 0.7 nm, 1.2 nm, and 2.7 nm in pore size distribution, indicating their hierarchical micromesopore structures (Figure 5(i)). In contrast, 3-CoZnNC and 7-CoZnNC only showed one peak at 1.2 nm which implied their microporous structure. By comparing these four derivatives, it was concluded that ZIF-L-Co derivative had a micromesopore structure with nanoparticles inside while ZIF-L-Zn derivative possessed a microporous structure with no nanoparticles, which was similar to the reported literatures [43, 44]. During the thermal treatment process, the 2-MeIM were decomposed and released reductive gases (NH_3_ and H_2_), which facilitated the formation of cobalt-based nanoparticles in ZIF-L-Co derivative [45]. Simultaneously, the cobalt-based nanoparticles exhibited high catalytic activity in ligand decomposition, leading to the mesopores around nanoparticles in ZIF-L-Co derivative [45]. It rendered 1-CoCN and 5-CoZnCN mesoporous structures and increments of the specific surface areas. On the contrary, the reductive gases were unable to reduce zinc ions at the thermal treatment temperature, resulting in the absence of either nanoparticles or mesoporous structure in ZIF-L-Zn derivative. Furthermore, under the influence of the thermal shrinkage effect during the MOF thermal treatment, the outer ZIF-L-Zn derivative may restrain the formation of the inner mesoporous structure. This might be accountable for the smaller specific surface area of 3-CoZnCN and 7-CoZnCN without mesopores [46]. Therefore, the properties of multilayer ZIF-L heterostructure derivatives including morphology, components, porosity, and functional nanoparticle distribution were possibly regulated by the selection of the outermost layers between two modular derivatives (ZIF-L-Co and ZIF-L-Zn derivatives). In other words, the two types of modular derivatives with distinctive properties had the ability to program an integrated heterostructure derivative according to the specific structural, compositional, and functional demands.

### 2.4. Magnetic Control and Selective Catalysis

**Figure 6:**
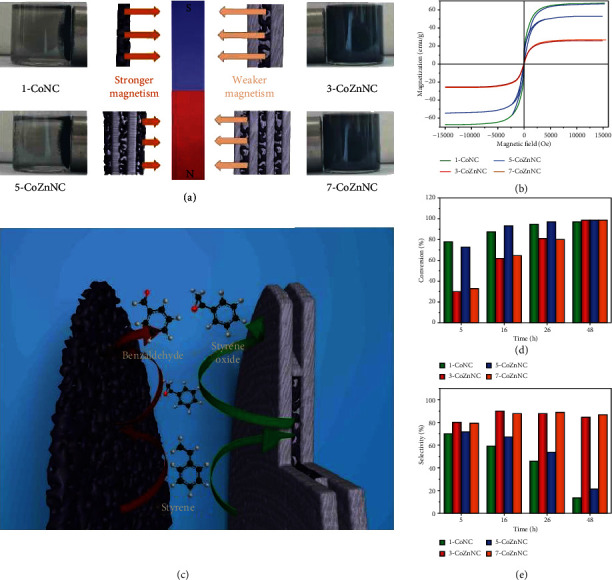
Magnetic and catalytic properties. (a) Schematic illustration of magnetism mechanism with magnet attraction photos at 40 seconds and (b) magnetic-field dependence of magnetization images measured at 300 K of the multilayer ZIF-L heterostructure derivatives. (c) Schematic illustration, (d) conversion, and (e) selectivity of the styrene epoxidation of the multilayer ZIF-L heterostructure derivatives.

Featuring adjustable compositions and structures of multilayer ZIF-L heterostructure derivatives, the derivative magnetisms with potential application prospects in biomedicine [47] and energy storage [48] are expected to be controlled by regulating cobalt-based nanoparticles. Specifically, 1-CoNC and 5-CoZnNC were easily attracted to the magnet within a short time while 3-CoZnNC and 7-CoZnNC showed relatively weaker magnetization, respectively, in acetonitrile (Figure 6(a)). The phenomenon has been further demonstrated by the field-dependent magnetization measurements (Figure 6(b)), where the saturated magnetization values of these four derivatives were 67.20, 26.42, 53.61, and 27.17 emu·g^−1^, respectively. The derivatives (1-CoNC and 5-CoZnNC) exhibited stronger magnetizations than the other two (3-CoZnNC and 7-CoZnNC), probably due to the space distribution of cobalt-based nanoparticles, which were distributed on the surface layers of 1-CoNC and 5-CoZnNC but were encapsulated inside layers of 3-CoZnNC and 7-CoZnNC. Although 1-CoNC had similar space distribution of cobalt-based nanoparticles compared with the 5-CoZnNC, it still showed higher saturated magnetization than 5-CoZnNC, mainly owing to the higher proportion of cobalt-based nanoparticles. In terms of 7-CoZnNC and 3-CoZnNC, the obtained results were similar. The proposed anisotropic MOF-on-MOF growth strategy may have potential to adjust magnetisms of ZIF-L heterostructure derivatives.

Usually, the structural design of MOFs and their derivatives has a significant impact on their catalytic performances [49–51]. Here, in order to further demonstrate the advantages of the synthesized multilayer ZIF-L heterostructure derivatives, the styrene epoxidation was chosen as the demo reaction for studying relationship between structures and catalytic performances. As a main product of styrene epoxidation, styrene oxide is an important chemical with wide applications in perfumery, petroleum, and medicine [52]. However, troubled by excessive by-products in the styrene epoxidation, there is an actual pursuit of finding suitable catalysts with superior styrene oxide selectivity [53, 54]. Specifically, a range of multilayer ZIF-L heterostructure derivatives were used as styrene epoxidation catalysts with styrene as the reactant, tert-butyl hydroperoxide (TBHP) as the oxidant, and acetonitrile as the solvent at 70°C for 48 h. We monitored the reaction process, respectively, at 5, 16, 26, and 48 h and took styrene oxide as the target product to calculate selectivity (Table S1). At 5 h, 1-CoNC and 5-CoZnNC showed higher conversion (78.1% and 73.0%) than 3-CoZnNC and 7-CoZnNC (29.4% and 32.7%), which could be attributed to the hierarchical micromesoporous structures and the more exposed cobalt-based nanoparticles of 1-CoNC and 5-CoZnNC for more efficient mass transfer (Figure 6(d)). Subsequently, the four catalysts showed improvement in conversion (almost 100%) at 48 h. At the same time, 1-CoNC and 5-CoZnNC had a slightly lower selectivity (70.4% and 72.5%) than 3-CoZnNC and 7-CoZnNC (80.6% and 79.0%) at 5 h (Figure 6(e)). However, as the reaction continued, the selectivity of 1-CoNC and 5-CoZnNC presented a decreasing tendency from 16 h (59.8% and 67.2%) to 48 h (13.4% and 21.2%). On the contrary, the selectivity of 3-CoZnNC and 7-CoZnNC raised up at 16 h (90.0% and 88.2%) and well maintained at 48 h (85.1% and 87.0%). The good selectivity of 3-CoZnNC and 7-CoZnNC was possibly attributed to the effect of unique structures including the outer ZIF-L-Zn derivative layer protection and microporous environment of cobalt-based nanoparticles for preventing further oxidation (Figure 6(c)). Significantly, the integration of the two modular derivatives could control the distribution, pore environment, and chemical state of catalytic active sites in these heterostructure derivatives, providing insights that can be useful in designing new catalysts with better performance.

## 3. Discussion

In summary, an anisotropic MOF-on-MOF growth strategy was developed between two isostructural MOFs (ZIF-L-Co and ZIF-L-Zn) by utilizing the intrinsic crystal surface discrepancies including coordination modes, relaxation effects, and surface energies. A range of multilayer sandwich-like zinc-cobalt alternating ZIF-L heterostructures such as ZIF-L-3, ZIF-L-5, and ZIF-L-7 were successfully prepared by multiple utilizations of such growth for controlling the specific growth rates and orders in different directions. These multilayer ZIF-L heterostructures exhibited advantages in controllable layer number, growth orientation, and spatial component distribution, which were favourable for derivative preparation. Furthermore, ZIF-L-Co and ZIF-L-Zn derivatives could be modular building blocks for designing multilayer ZIF-L heterostructure derivatives with hierarchical porosity and tunable distribution of cobalt-based nanoparticles, which exhibited fascinating performance in applications such as magnetism and styrene epoxidation. The proposed concept not only is suitable to the template ZIF-L, but also offers a prospect to more template MOFs with intrinsic crystal surface discrepancies for MOF heterostructures by anisotropic MOF-on-MOF growth.

## 4. Materials and Methods

### 4.1. Materials and Measurements

All commercially available reagents and solvents were used as received without further purification. Cobalt nitrate hexahydrate (98%), zinc nitrate hexahydrate (>99%), 2-methylimidazole (99%), acetonitrile (>99%), and styrene (>90%) were bought from Sigma-Aldrich. Tert-butyl hydroperoxide (TBHP 70%) was bought from Acros Organics. XRD patterns of samples were recorded with a Bruker AXS D8 Advance diffractometer using nickel-filtered Cu K*α* radiation (*λ* = 1.5406 Å). SEM images were taken by a JEOL JSM-7600 with an accelerating voltage of 5 kV. TEM images were taken by JEOL JEM-2100 Plus at an accelerating voltage of 200 kV. Thermogravimetric analysis was performed on a Q500 TGA (TA Instruments) under nitrogen gas flow at 5°C·min^−1^ from 30°C to 800°C. Nitrogen adsorption–desorption isotherms of powder samples were measured with Micromeritics ASAP 2460 adsorption apparatus at 77 K up to 1 bar. Before starting the adsorption measurements, each sample was activated by heating under vacuum at 120°C for 12 h. The pore textural properties including Brunauer–Emmett–Teller surface area, pore volume, and pore size were obtained by analyzing nitrogen adsorption–desorption isotherms with density functional theory (DFT) method. Gas chromatography (GC) spectra were recorded on Agilent Technologies 7890B. Vibrating sample magnetometer (VSM) was used for field-dependent magnetization measurements from −15000 Oe to 15000 Oe at room temperature.

### 4.2. Preparation of ZIF-L-Co

It was synthesized according to a reported method with minor modifications [30]. 116.4 mg cobalt nitrate hexahydrate was dissolved in 4 mL of deionized water. 262.7 mg 2-methylimidazole was dissolved in 5 mL deionized water. The two solutions were mixed under stirring at room temperature for 2 h. The product was centrifuged at 8000 rpm for 5 min (product A). Finally, product A was washed 3 times with ethanol, dried at 70°C, and stored in a vacuum desiccator.

### 4.3. Preparation of ZIF-L-3

The product A was ultrasonic dispersed in 15 mL aqueous solution containing 390 mg 2-methylimidazole for about 3 min. 15 mL aqueous solution containing 177 mg zinc nitrate hexahydrate was added into the solution under stirring at room temperature for 1 h. The product was centrifuged at 8000 rpm for 5 min (product B). Finally, product B was washed 3 times with ethanol, dried at 70°C, and stored in a vacuum desiccator.

### 4.4. Preparation of ZIF-L-5

The product B was ultrasonic dispersed in 15 mL aqueous solution containing 390 mg 2-methylimidazole for about 3 min. 15 mL aqueous solution containing 177 mg cobalt nitrate hexahydrate was added into the solution under stirring at room temperature for 1 h. The product was centrifuged at 8000 rpm for 5 min (product C). Finally, product C was washed 3 times with ethanol, dried at 70°C, and stored in a vacuum desiccator.

### 4.5. Preparation of ZIF-L-7

The product C was ultrasonic dispersed in 15 mL aqueous solution containing 177 mg zinc nitrate hexahydrate was added into the solution under stirring at room temperature for 1 h. The product was centrifuged at 8000 rpm for 5 min (product D). Finally, product D was washed 3 times with ethanol, dried at 70°C, and stored in a vacuum desiccator.

### 4.6. Preparation of ZIF-L-Zn

It was synthesized according to a reported method with minor modifications [31]. 120 mg zinc nitrate hexahydrate was dissolved in 8 mL of deionized water. 260 mg 2-methylimidazole was dissolved in 8 mL deionized water. The two solutions were mixed under stirring at room temperature for 2 h. The product was centrifuged at 8000 rpm for 5 min (product E). Finally, product E was washed 3 times with ethanol, dried at 70°C, and stored in a vacuum desiccator.

### 4.7. Preparation of ZIF-L-Zn-Based Three-Layer Heterostructure

The product E was ultrasonic dispersed in 15 mL aqueous solution containing 390 mg 2-methylimidazole for about 3 min. 15 mL aqueous solution containing 177 mg cobalt nitrate hexahydrate was added into the solution under stirring at room temperature for 1 h. The product was centrifuged at 8000 rpm for 5 min and washed 3 times with ethanol, dried at 70°C, and stored in a vacuum desiccator.

### 4.8. Preparation of Multilayer ZIF-L Heterostructure Derivatives

The multilayer ZIF-L heterostructures including ZIF-L-Co, ZIF-L-3, ZIF-L-5, and ZIF-L-7 were calcined in a tube furnace in nitrogen with the temperature increasing from 30°C to 130°C for 60 min, 130°C to 280°C for 75 min, 280°C to 500°C for 22 min, and 500°C to 600°C for 20 min and held at 600°C for 120 min. The multilayer ZIF-L heterostructure derivatives including 1-CoNC, 3-CoZnNC, 5-CoZnNC, and 7-CoZnNC were obtained after being cooled to room temperature.

### 4.9. Styrene Epoxidation

Styrene (0.1 mL), acetonitrile (1 mL), TBHP (0.5 mL), and catalysts (10 mg 1-CoNC, 30 mg 3-CoZnNC, 16.7 mg 5-CoZnNC, and 17.5 mg 7-CoZnNC according to the percentage of Co-NC layers) were introduced into a 10 mL vial in an enclosed environment. The mixture was stirred at 70°C for 48 h, and the reaction products were directly monitored by GC.

## Data Availability

All data needed to evaluate the conclusions in the paper are present in the paper and the Supplementary Materials. Additional data related to this paper may be requested from the authors.

## Supplementary Materials

Supplementary Materials Supplementary 1. Figure S1: SEM images of the series of multilayer ZIF-L. (a) ZIF-L-Co, (b) ZIF-L-Zn, (c) ZIF-L-3, (d) ZIF-L-5, (e) ZIF-L-7, and (f) ZIF-L-9. Supplementary 2. Figure S2: elemental mapping of ZIF-L-3. Supplementary 3. Figure S3: elemental mapping of ZIF-L-5. Supplementary 4. Figure S4: TEM images of ZIF-L-3 in the formation process at (a) 10 s, (b) 30 s, (c) 1 min, (d) 3 min, (e) 5 min, and (f) 10 min. Supplementary 5. Figure S5: schematic diagram of coordination on different crystal surfaces of ZIF-L-Zn. Supplementary 6. Figure S6: TEM images of ZIF-L-Zn-based three-layer heterostructure. Supplementary 7. Figure S7: elemental mapping of ZIF-L-Zn-based three-layer heterostructure. Supplementary 8. Figure S8: SEM images of ZIF-L-Zn-based three-layer heterostructure in the formation process at (a) 10 s, (b) 1 min, (c) 5 min, and (d) 10 min. Supplementary 9. Figure S9: TEM images of ZIF-L-Zn-based three-layer heterostructure in the formation process at (a) 10 s, (b) 1 min, (c) 5 min, and (d) 10 min. Supplementary 10. Figure S10: SEM images of multilayer ZIF-L heterostructure derivatives. (a) 1-CoNC, (b) 3-CoZnNC, (c) 5-CoZnNC, and (d) 7-CoZnNC. Supplementary 11. Figure S11: PXRD patterns of multilayer ZIF-L heterostructure derivatives. Supplementary 12. Figure S12: the 1-CoNC XPS spectra of (a) survey spectrum, (b) C 1s spectrum, and (c) N 1*s* spectrum. The 3-CoZnNC XPS spectra of (d) survey spectrum, (e) C 1*s* spectrum, (f) N 1*s* spectrum, (g) Co 2*p* spectrum, and (h) Zn 2*p* spectrum. Supplementary 13. Figure S13: the line scan and elemental mapping of 3-CoZnNC. Supplementary 14. Table S1: catalytic properties in styrene epoxidation.
