# Supplementary material for: Anisotropic MOF-on-MOF Growth of Isostructural Multilayer Metal–Organic Framework Heterostructures
Source: Research (Wash D C). 2021 Nov 16;2021:9854946. doi: 10.34133/2021/9854946 (PMC8613540; doi:10.34133/2021/9854946)
Supplement: Supplementary Materials — Supplementary 1. Figure S1: SEM images of the series of multilayer ZIF-L. (a) ZIF-L-Co, (b) ZIF-L-Zn, (c) ZIF-L-3, (d) ZIF-L-5, (e) ZIF-L-7, and (f) ZIF-L-9. Supplementary 2. Figure S2: elemental mapping of ZIF-L-3. Supplementary 3. Figure S3: elemental mapping of ZIF-L-5. Supplementary 4. Figure S4: TEM images of ZIF-L-3 in the formation process at (a) 10 s, (b) 30 s, (c) 1 min, (d) 3 min, (e) 5 min, and (f) 10 min. Supplementary 5. Figure S5: schematic diagram of coordination on different crystal surfaces of ZIF-L-Zn. Supplementary 6. Figure S6: TEM images of ZIF-L-Zn-based three-layer heterostructure. Supplementary 7. Figure S7: elemental mapping of ZIF-L-Zn-based three-layer heterostructure. Supplementary 8. Figure S8: SEM images of ZIF-L-Zn-based three-layer heterostructure in the formation process at (a) 10 s, (b) 1 min, (c) 5 min, and (d) 10 min. Supplementary 9. Figure S9: TEM images of ZIF-L-Zn-based three-layer heterostructure in the formation process at (a) 10 s, (b) 1 min, (c) 5 min, and (d) 10 min. Supplementary 10. Figure S10: SEM images of multilayer ZIF-L heterostructure derivatives. (a) 1-CoNC, (b) 3-CoZnNC, (c) 5-CoZnNC, and (d) 7-CoZnNC. Supplementary 11. Figure S11: PXRD patterns of multilayer ZIF-L heterostructure derivatives. Supplementary 12. Figure S12: the 1-CoNC XPS spectra of (a) survey spectrum, (b) C 1s spectrum, and (c) N 1s spectrum. The 3-CoZnNC XPS spectra of (d) survey spectrum, (e) C 1s spectrum, (f) N 1s spectrum, (g) Co 2p spectrum, and (h) Zn 2p spectrum. Supplementary 13. Figure S13: the line scan and elemental mapping of 3-CoZnNC. Supplementary 14. Table S1: catalytic properties in styrene epoxidation. [file 9854946.f1.docx]

**Supplementary Materials**

**Anisotropic MOF-on-MOF Growth of Isostructural Multilayer Metal–Organic Framework Heterostructures**

Zhida Gu^1^, Wenlei Zhang^1^, Ting Pan^2^, Yu Shen^2^, Peishan Qin^2^, Peng Zhang^2^, Xiaohan Li^1^, Liwei Liu^2^, Linjie Li^1^, Yu Fu^1,⋆^, Weina Zhang^2,⋆^, and Fengwei Huo^2,⋆^

^1^College of Science, Northeastern University, Shenyang 100819, China

^2^Key Laboratory of Flexible Electronics (KLOFE), Institute of Advanced Materials (IAM), Nanjing Tech University (NanjingTech), Nanjing 211800, China

^⋆^Correspondence should be addressed to Yu Fu; fuyu@mail.neu.edu.cn, Weina Zhang; iamwnzhang@njtech.edu.cn, Fengwei Huo; iamfwhuo@njtech.edu.cn

Table of Contents

Figure S1. SEM images of the serie of multi-layer ZIF-L. (a) ZIF-L-Co, (b) ZIF-L-Zn, (c) ZIF-L-3, (d) ZIF-L-5, (e) ZIF-L-7 and (f) ZIF-L-9.

Figure S2. Elemental mapping of ZIF-L-3.

Figure S3. Elemental mapping of ZIF-L-5.

Figure S4. TEM images of ZIF-L-3 in the formation process at (a) 10 s, (b) 30 s, (c) 1 min, (d) 3 min, (e) 5 min and (f) 10 min.

Figure S5. Schematic diagram of coordination on different crystal surfaces of ZIF-L-Zn.

Figure S6. TEM images of ZIF-L-Zn based three-layer heterostructure.

Figure S7. Elemental mapping of ZIF-L-Zn based three-layer heterostructure.

Figure S8. SEM images of ZIF-L-Zn based three-layer heterostructure in the formation process at (a) 10 s, (b) 1 min, (c) 5 min and (d) 10 min.

Figure S9. TEM images of ZIF-L-Zn based three-layer heterostructure in the formation process at (a) 10 s, (b) 1 min, (c) 5 min and (d) 10 min.

Figure S10. SEM images of multilayer ZIF-L heterostructure derivatives. (a) 1-CoNC, (b) 3-CoZnNC, (c) 5-CoZnNC and (d) 7-CoZnNC.

Figure S11. PXRD patterns of multi-layer ZIF-L heterostructure derivatives.

Figure S12. The 1-CoNC XPS spectra of (a) survey spectrum, (b) C 1*s* spectrum and (c) N 1*s* spectrum. The 3-CoZnNC XPS spectra of (d) survey spectrum, (e) C 1*s* spectrum, (f) N 1*s* spectrum, (g) Co 2*p* spectrum and (h) Zn 2*p* spectrum.

Figure S13. The line scan and elemental mapping of 3-CoZnNC.

Table S1. Catalytic properties in styrene epoxidation.

**
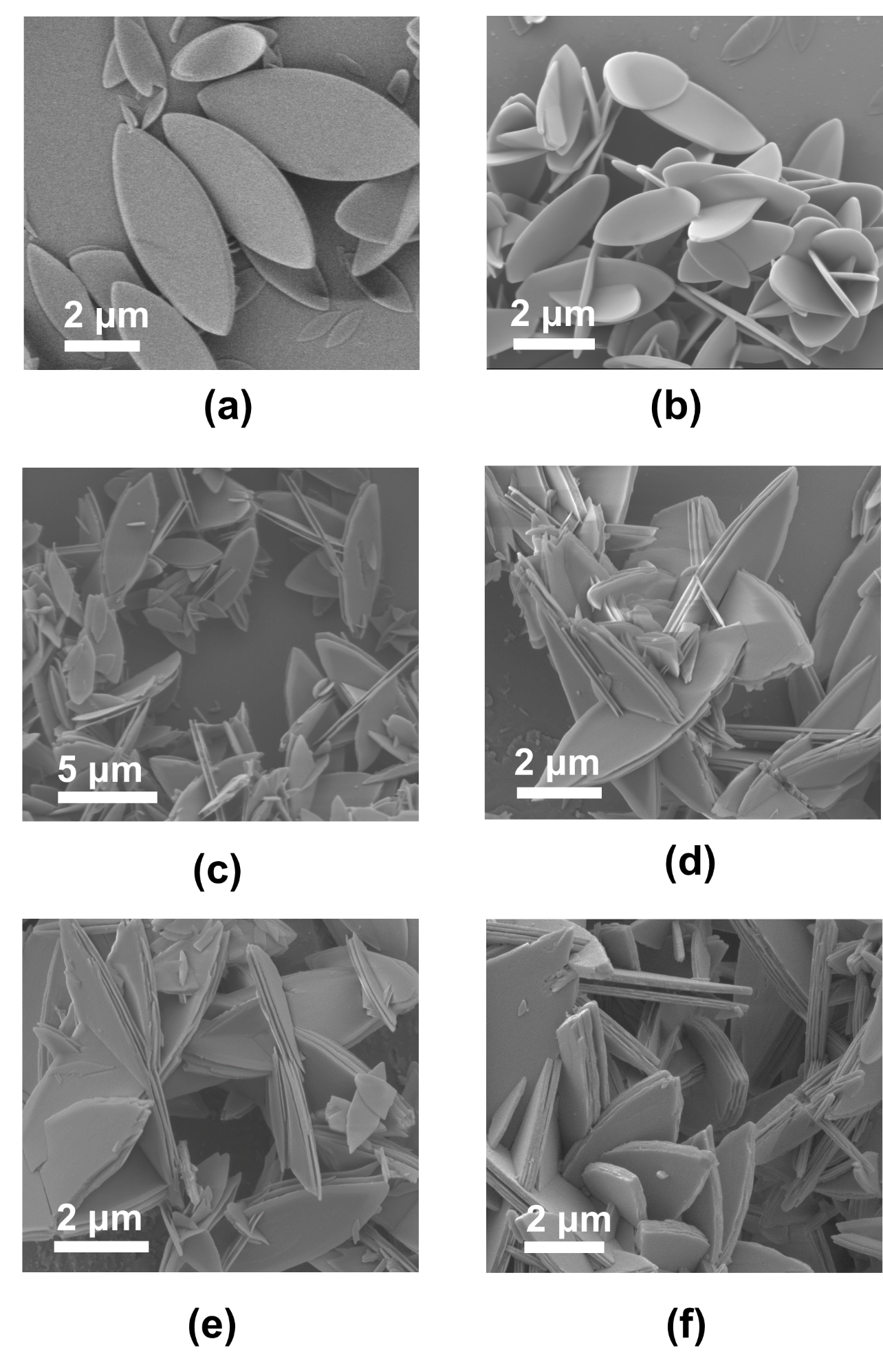
**

Figure S1. SEM images of the serie of multi-layer ZIF-L. (a) ZIF-L-Co, (b) ZIF-L-Zn, (c) ZIF-L-3, (d) ZIF-L-5, (e) ZIF-L-7 and (f) ZIF-L-9.


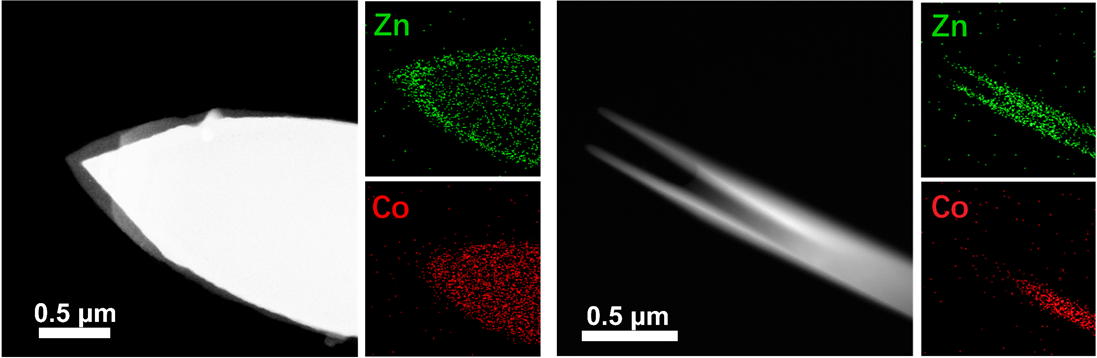


Figure S2. Elemental mapping of ZIF-L-3.


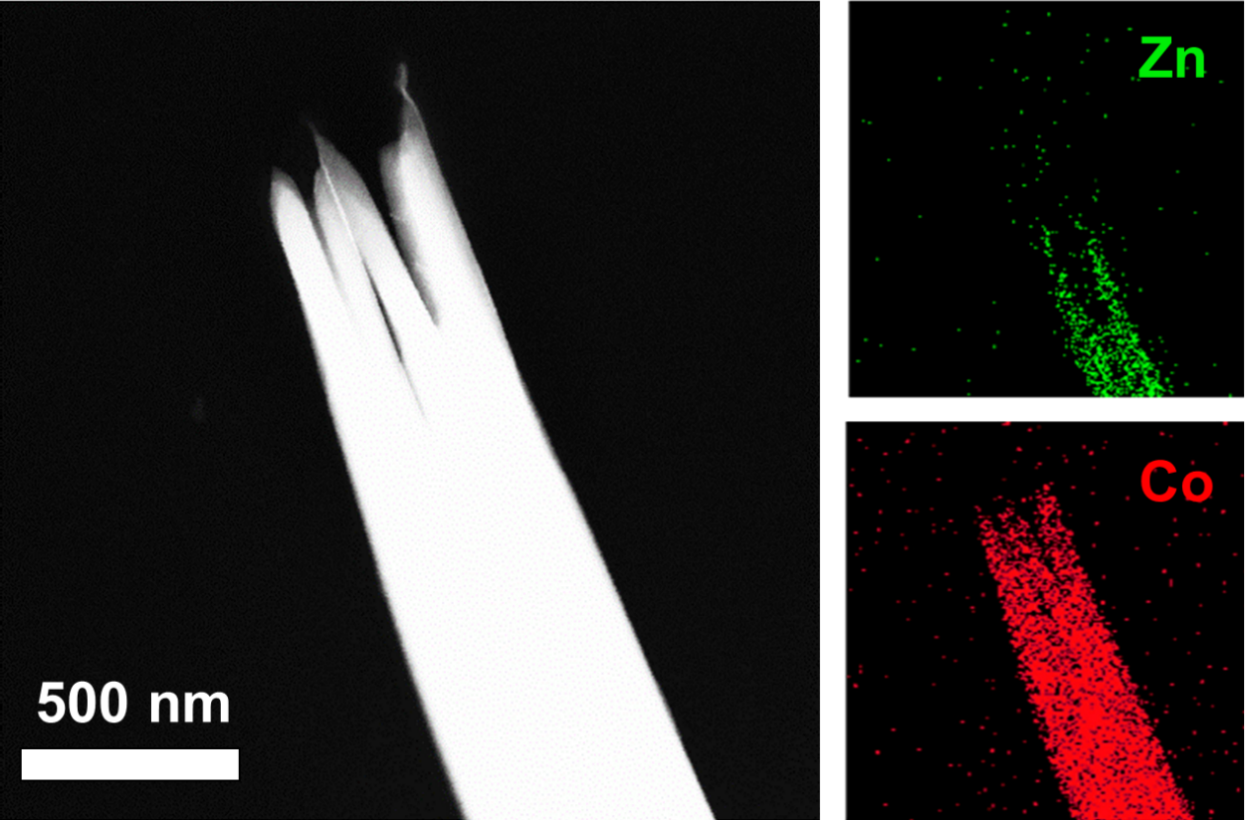


Figure S3. Elemental mapping of ZIF-L-5.


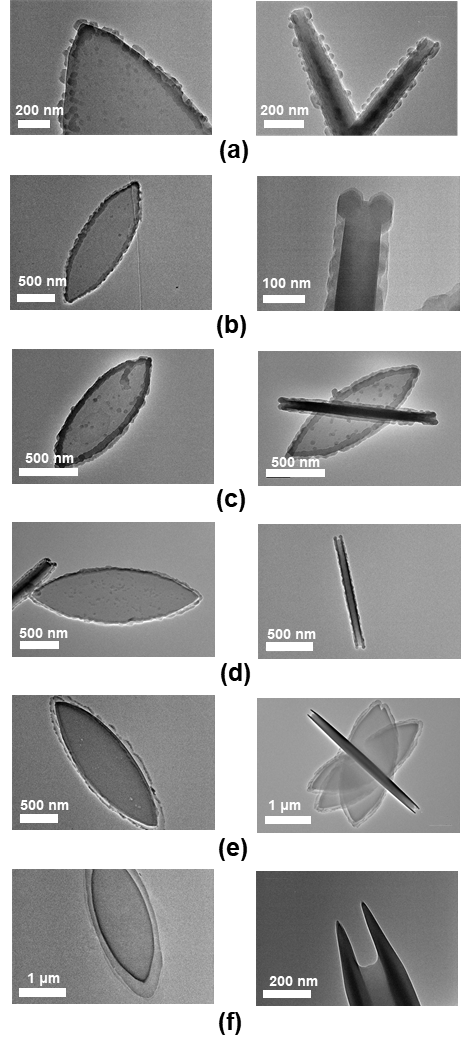


Figure S4. TEM images of ZIF-L-3 in the formation process at (a) 10 s, (b) 30 s, (c) 1 min, (d) 3 min, (e) 5 min and (f) 10 min.

**
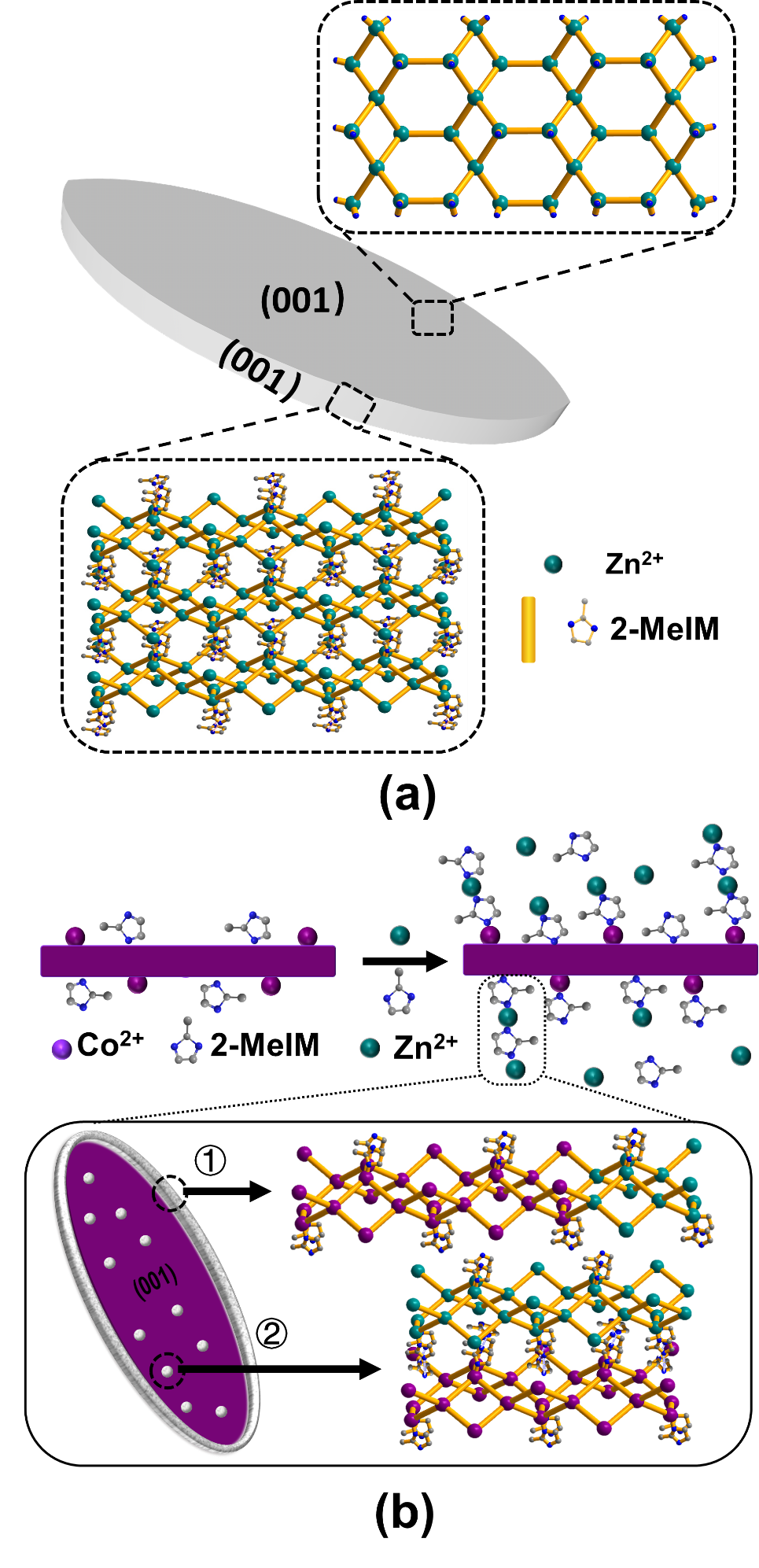
**

Figure S5. Schematic diagram of coordination on different crystal surfaces of ZIF-L-Zn.


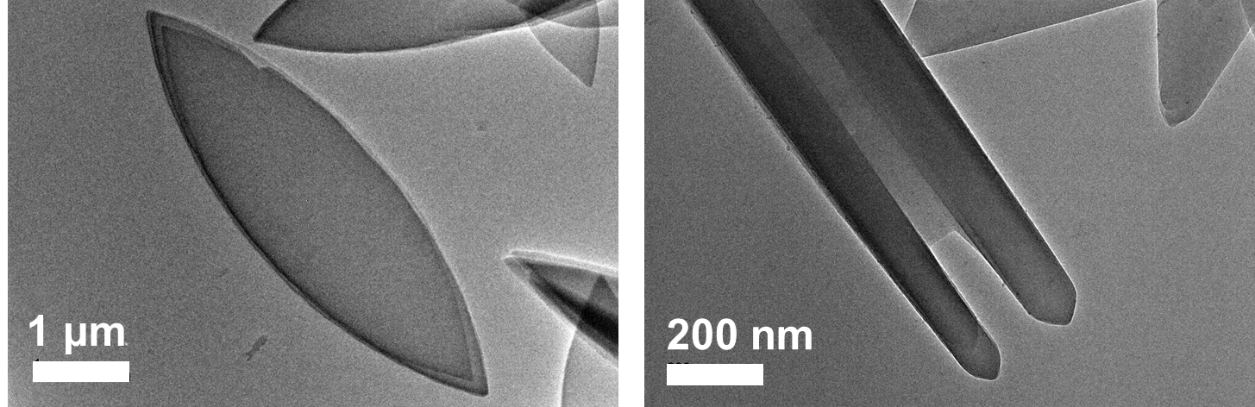


Figure S6. TEM images of ZIF-L-Zn based three-layer heterostructure.


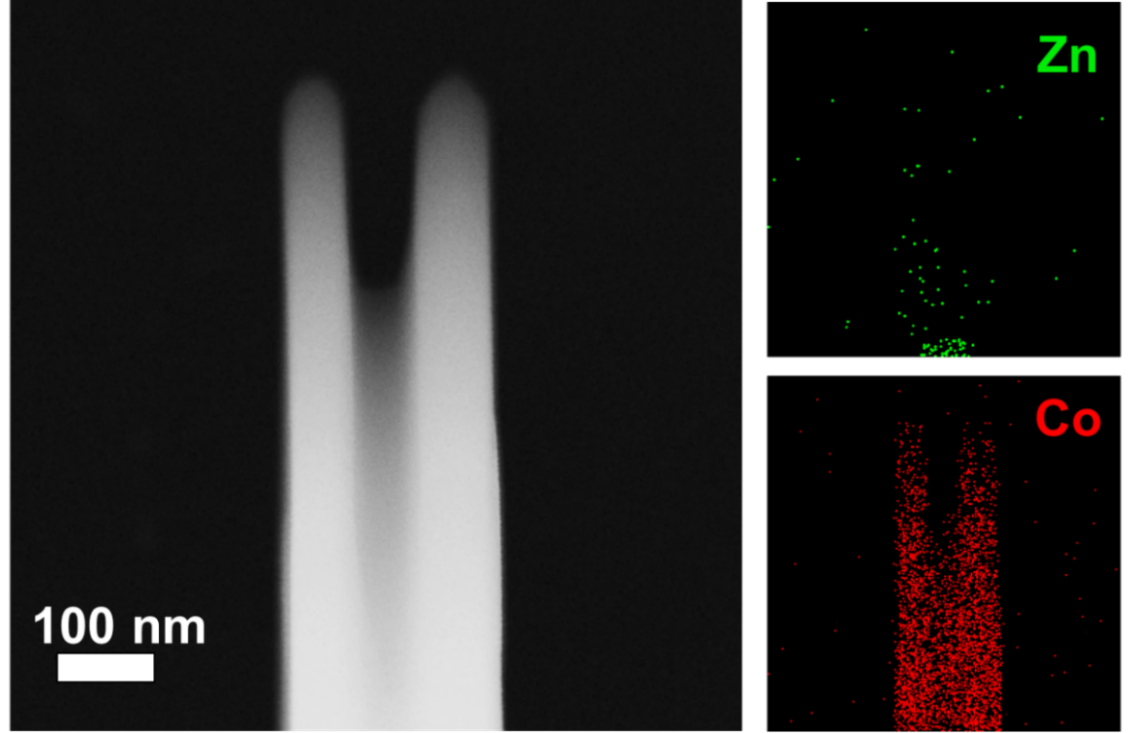


Figure S7. Elemental mapping of ZIF-L-Zn based three-layer heterostructure.


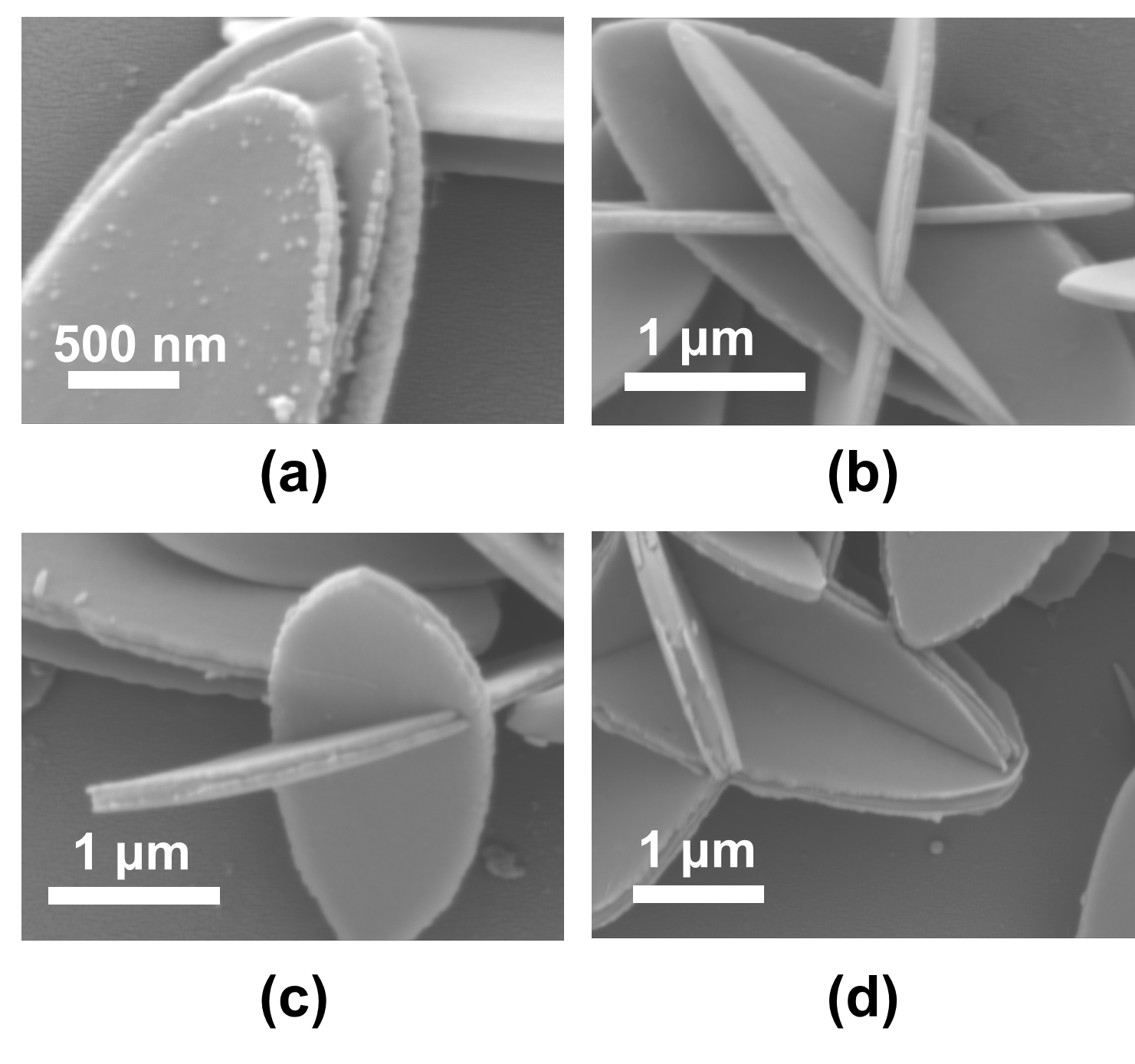


Figure S8. SEM images of ZIF-L-Zn based three-layer heterostructure in the formation process at (a) 10 s, (b) 1 min, (c) 5 min and (d) 10 min.


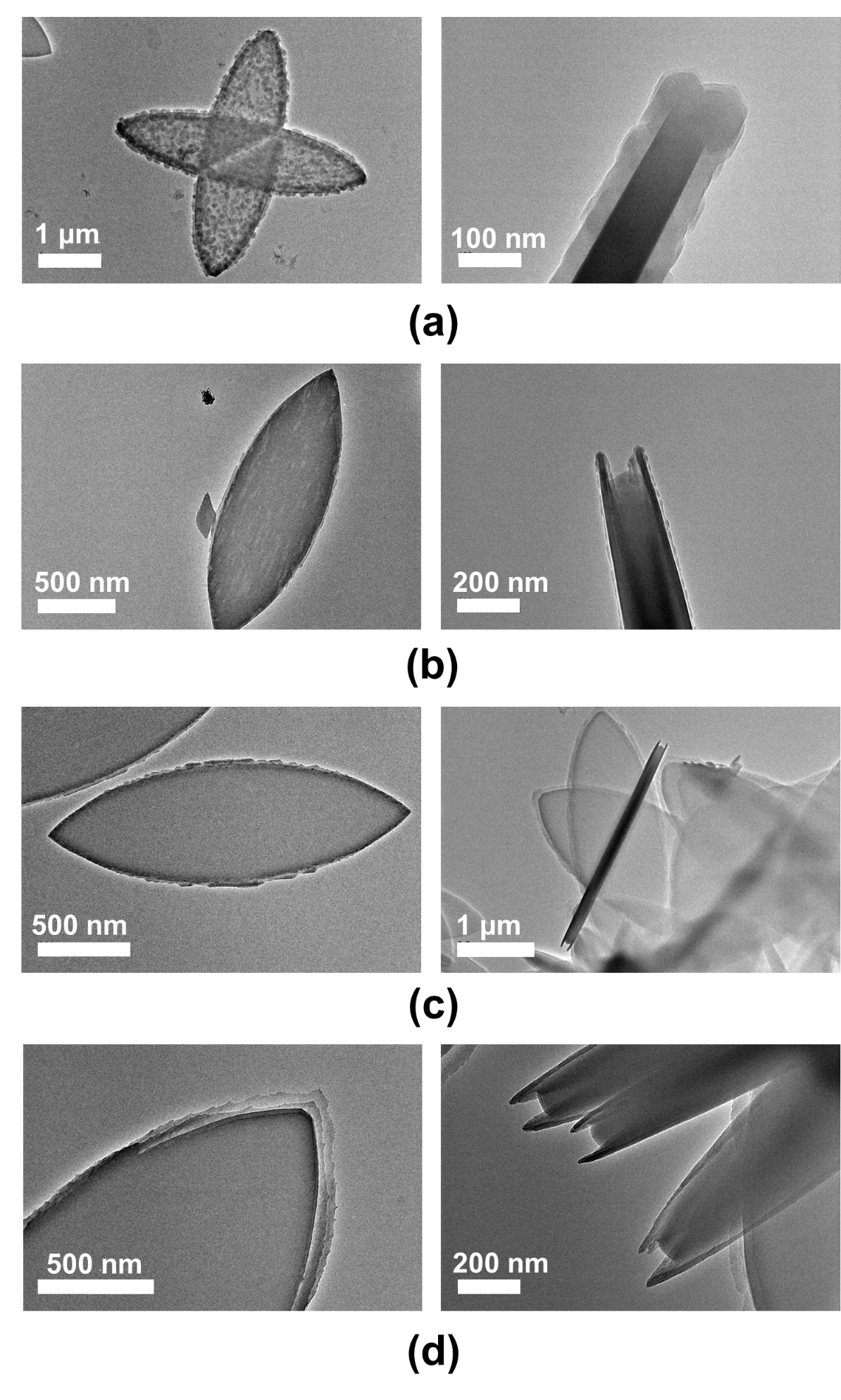


Figure S9. TEM images of ZIF-L-Zn based three-layer heterostructure in the formation process at (a) 10 s, (b) 1 min, (c) 5 min and (d) 10 min.


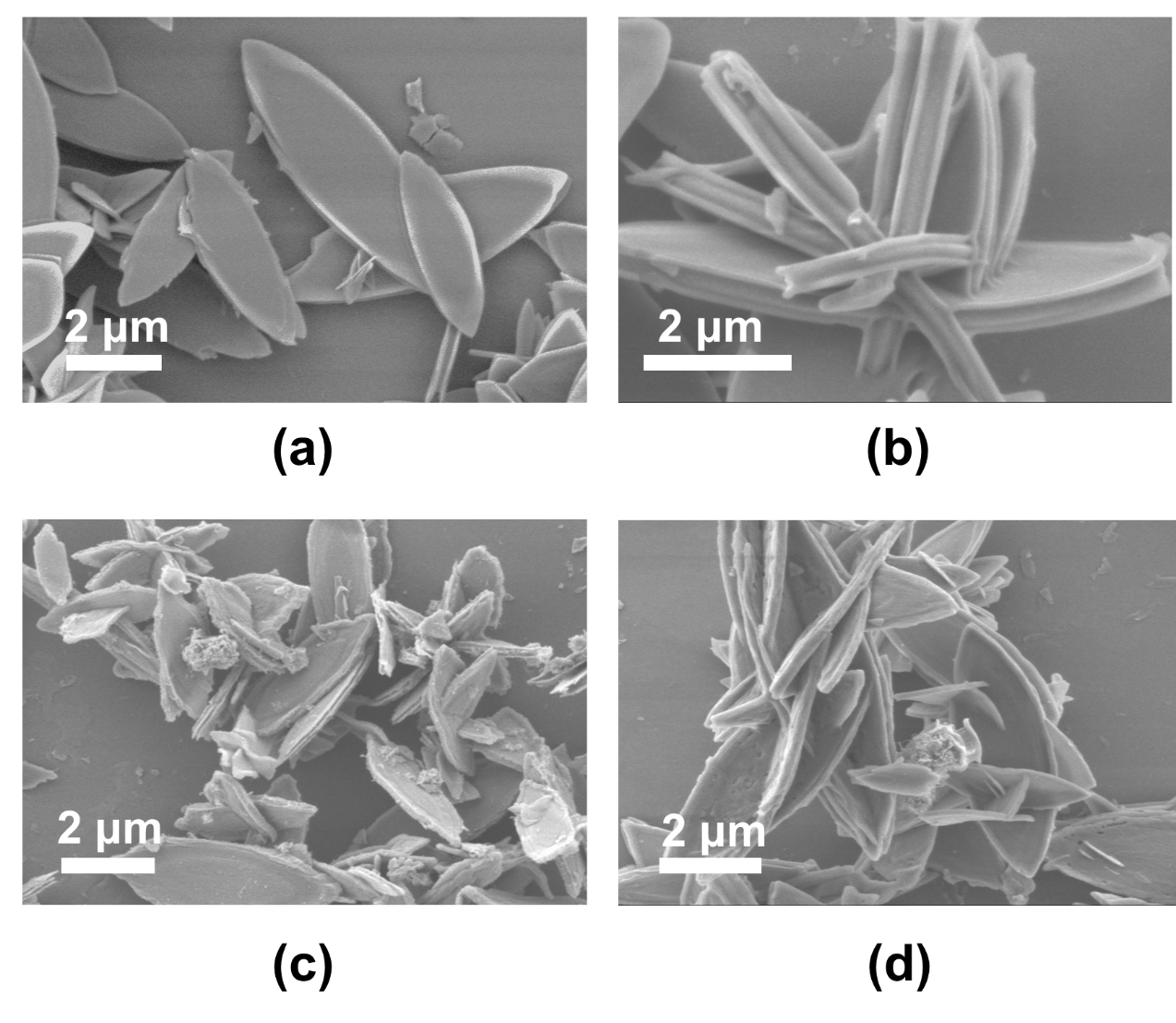


Figure S10. SEM images of multilayer ZIF-L heterostructure derivatives. (a) 1-CoNC, (b) 3-CoZnNC, (c) 5-CoZnNC and (d) 7-CoZnNC.


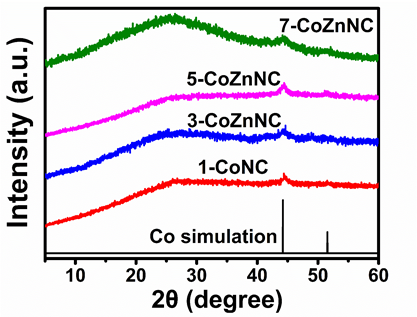


Figure S11. PXRD patterns of multi-layer ZIF-L heterostructure derivatives.


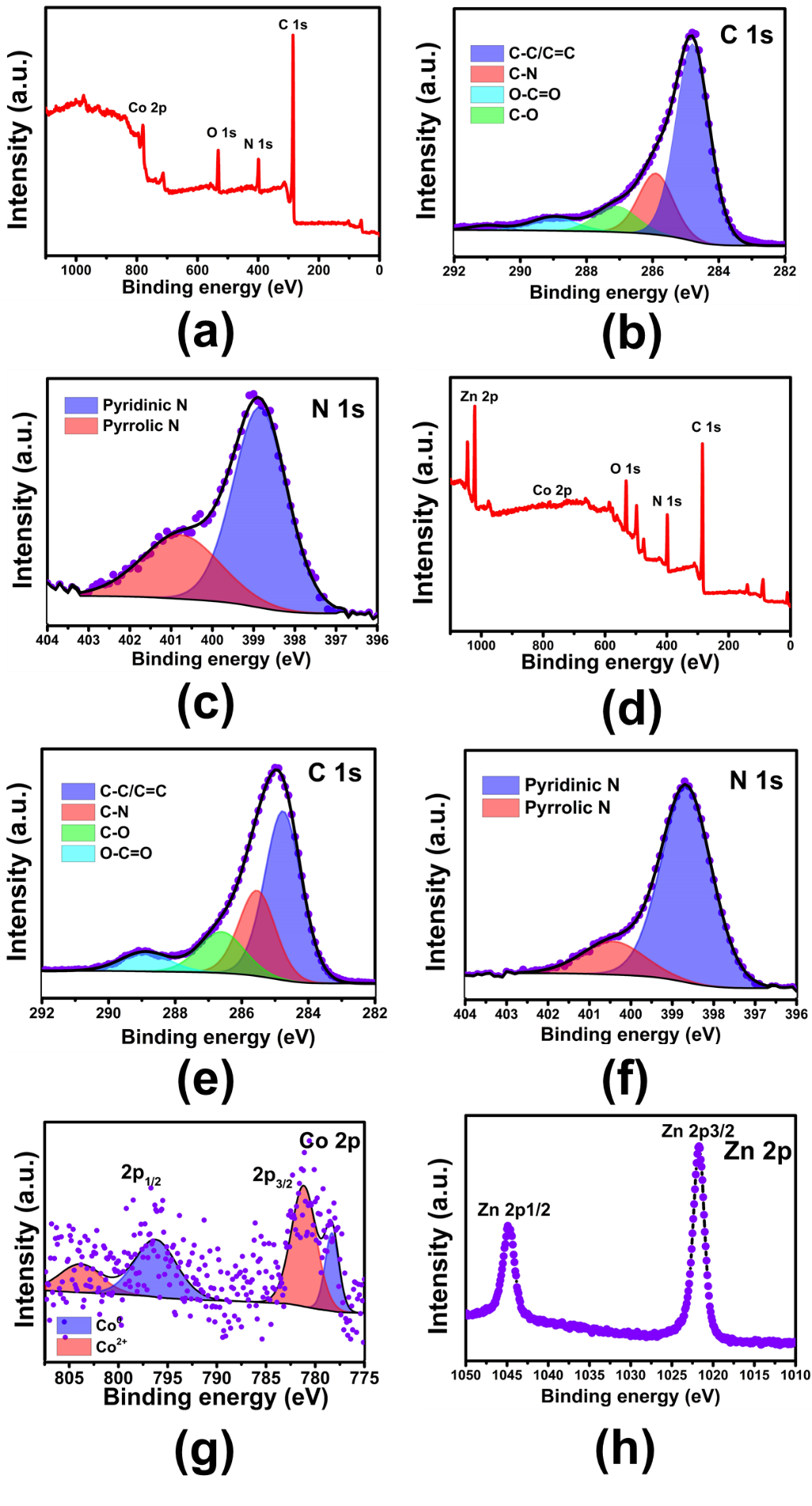


Figure S12. The 1-CoNC XPS spectra of (a) survey spectrum, (b) C 1*s* spectrum and (c) N 1*s* spectrum.

The 1-CoNC XPS spectra of (d) survey spectrum, (e) C 1*s* spectrum, (f) N 1*s* spectrum, (g) Co 2p spectrum and (h) Zn 2*p* spectrum.


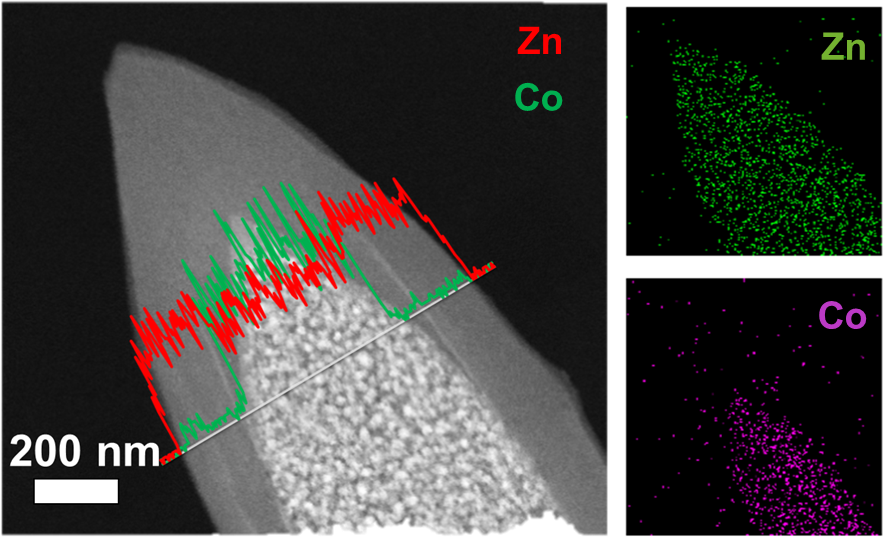


Figure S13. The line scan and elemental mapping of 3-CoZnNC.

Table S1. Catalytic properties in styrene epoxidation.

| Catalysts | Times(h) | Conv. (%) | Sel. (%) |
| --- | --- | --- | --- |
| 1-CoNC | 5  16  26  48 | 78.13  87.59  95.55  97.22 | 70.42  59.77  45.91  13.43 |
| 3-CoZnNC | 5  16  26  48 | 29.45  62.45  81.33  98.90 | 80.60  90.01  87.47  85.10 |
| 5-CoZnNC | 5  16  26  48 | 72.98  93.35  97.27  99.12 | 72.45  67.17  53.68  21.21 |
| 7-CoZnNC | 5  16  26  48 | 32.66  65.33  80.68  98.83 | 78.99  88.20  89.20  87.00 |
